# Evaluation of a phytogenic blend supplementation on reproductive performance, antioxidant status, and immune function in broiler breeder roosters

**DOI:** 10.1016/j.psj.2025.105825

**Published:** 2025-09-10

**Authors:** Morteza Asghari-Moghadam, Hamid-Reza Behboodi, Mehran Mehri

**Affiliations:** a Department of Animal Sciences, Faculty of Agriculture, University of Zabol; Sistan, 98613-35856, Iran; b Department of Animal and Poultry Physiology, Faculty of Animal Science, Gorgan University of Agricultural Sciences and Natural Resources, Gorgan, 49138-15739, Iran

**Keywords:** Broiler breeder roosters, LibidoMax, Reproductive hormones, Antioxidant, Immune response

## Abstract

Rooster fertility declines with aging, marked by reduced testicular weight, testosterone, semen quality, and antioxidant capacity, leading to lower hatching egg production and economic loss. This study evaluated the physiological effects and optimal inclusion rate of LibidoMax, a phytogenic supplement, on reproductive hormones, semen quality, antioxidant status, immune function, and metabolic biomarkers in aged broiler breeder roosters, using broken-line regression modeling. Twenty roosters (42 weeks old) were randomly assigned to four treatments (0, 100, 200, or 300 mL LibidoMax per 100 kg feed) for 70 days, including 14 days of acclimation. LibidoMax significantly enhanced serum levels of gonadotropin-releasing hormone (GnRH), luteinizing hormone (LH), follicle-stimulating hormone (FSH), and testosterone (P < 0.001). Broken-line regressions identified optimal hormonal plateaus between 182–190 mL/100 kg feed. Semen volume, sperm motility, viability, and concentration improved linearly, with breakpoints around 200 mL/100 kg feed, while sperm abnormality was minimized at ∼179 mL/100 kg feed. Hematological parameters improved with supplementation. Hemoglobin (HGB) and hematocrit (HCT) reached breakpoints at 176.5 and 179.6 mL/100 kg feed, respectively. Red and white blood cell counts (RBC and WBC) plateaued at 178.7–184.1 mL/100 kg feed, indicating enhanced oxygen transport and immune activation. Antioxidant enzymes, including total antioxidant capacity (T-AOC), glutathione peroxidase (GPx), and superoxide dismutase (SOD), peaked at ∼200 mL/100 kg feed, while malondialdehyde (MDA), a lipid peroxidation marker, was minimized at 100–110 mL/100 kg feed, reflecting early saturation of oxidative stress reduction. Lipid profiles were favorably modulated: triglycerides (TG), low-density lipoprotein (LDL), very low-density lipoprotein (VLDL), and total cholesterol decreased (breakpoints: 169–178 mL/100 kg feed), whereas high-density lipoprotein (HDL) peaked at 182 mL/100 kg feed. Liver enzymes, alanine transaminase (ALT), aspartate transaminase (AST), and alkaline phosphatase (ALP), were reduced at 170–180 mL/100 kg feed, indicating improved hepatic function. Principal Component Analysis (PCA) confirmed multivariate separation among treatment groups, with LibidoMax positively associated with reproductive, antioxidant, and immune enhancements and negatively with hepatic stress and lipid markers. In conclusion, 180–200 mL/100 kg feed represents the optimal inclusion range of LibidoMax to improve reproductive efficiency, immunity, and systemic health in aged breeder roosters.

## Introduction

Rooster fertility declines after 40 weeks of age, marked by reduced testicular weight, testosterone, semen quality, and antioxidant capacity, leading to lower hatching egg production and economic loss (Barbarestani, et al., 2025). Nutraceuticals and functional feeds are increasingly recognized for their potential to enhance animal performance and health through natural feed-based interventions (Alagawany, et al., 2021). Their efficacy is linked to their ability to modulate key physiological pathways, such as hormonal regulation, metabolic homeostasis, immune responses, and antioxidant defense systems (Du, et al., 2022; Koyama, et al., 2021). By influencing these pathways, nutraceuticals can help establish normal physiological health, prevent diseases, and improve production performance in animals (El Basuini, et al., 2016). This shift towards nutraceuticals aligns with consumer preferences for sustainably produced animal products and reduced reliance on synthetic compounds or antibiotics, driven by concerns over antibiotic resistance and the desire for natural alternatives in animal husbandry (Alagawany, et al., 2021). Overall, nutraceuticals and functional feeds represent a promising approach to improving animal health and performance while supporting sustainable practices in the industry.

In male animals, reproductive success is significantly influenced by the interplay of hormones, particularly gonadotropin-releasing hormone (GnRH), luteinizing hormone (LH), and testosterone. GnRH is secreted by the hypothalamus and plays a crucial role in stimulating the pituitary gland to release LH and follicle-stimulating hormone (FSH). The pulsatile secretion of GnRH is essential for the regulation of reproductive processes, including spermatogenesis and the release of reproductive hormones (Zhao, et al., 2023). LH acts on Leydig cells in the testes to stimulate the production of testosterone, which is vital for spermatogenesis, libido, and the development of secondary sexual characteristics. Testosterone, in turn, influences various physiological processes, including sperm maturation and aggressive behavior (Bernard, et al., 2019). The quality of semen, which includes sperm count, motility, and morphology, is closely linked to the levels of testosterone and the balance of LH and FSH. Psychological stress can negatively impact these hormone levels, leading to decreased semen quality (Bhongade, et al., 2015). Semen quality is essential for male fertility, as high-quality semen increases the chances of successful fertilization and embryonic viability. However, oxidative stress, inflammation, and metabolic imbalances can significantly compromise sperm integrity through mechanisms such as DNA damage and lipid peroxidation. Aging (Asghari-Moghadam, et al., 2024) and oxidative stress arises from an imbalance between reactive oxygen species (ROS) and antioxidants, leading to sperm dysfunction in roosters (Agarwal, et al., 2016; Darbandi, et al., 2018; Virant-Klun, et al., 2022). Inflammation, often associated with infections, can elevate pro-inflammatory cytokines that disrupt spermatogenesis and contribute to oxidative stress (Agarwal, et al., 2003; Pasqualotto, et al., 2000), therefore, improving semen quality is particularly critical in species that rely on artificial insemination, as the success of these techniques is directly influenced by the quality of the semen used.

Total antioxidant capacity (T-AOC), superoxide dismutase (SOD), and glutathione peroxidase (GPx) play significant roles in influencing semen quality in roosters. Antioxidants mitigate oxidative stress, which otherwise compromises sperm integrity and function. Higher T-AOC levels in seminal plasma are associated with improved sperm motility and viability. For instance, dietary supplementation with ginger has been shown to enhance T-AOC, leading to better sperm forward motility and overall semen quality in aged roosters (Akhlaghi, et al., 2014; Nemati, et al., 2023). SOD is crucial for protecting sperm from oxidative damage. Studies indicate that increased SOD activity correlates positively with semen quality parameters such as sperm concentration and motility. For example, higher SOD levels were observed in roosters fed diets supplemented with antioxidants, which improved their semen quality (Murawski, et al., 2007; Ratchamak, et al., 2023). GPx also contributes to the antioxidant defense in sperm. Research has shown that GPx activity can be enhanced through dietary interventions, leading to reduced oxidative stress and improved sperm quality. For instance, the addition of antioxidants in freezing extenders has been linked to increased GPx activity, which in turn supports better post-thaw semen quality (Najafi, et al., 2019; Sun, et al., 2021).

LibidoMax is a proprietary blend comprising sage (*Salvia officinalis*), rose flower (*Rosa* spp.) extracts, flaxseed oil (rich in omega-3 fatty acids), vitamins E and C, selenium, and zinc, formulated to enhance reproductive performance, semen quality, antioxidant status, immune function, and metabolic health in broiler breeder roosters. Sage contains biologically active compounds such as carnosol and carnosic acid, which are well-known antioxidants with anti-inflammatory and antimicrobial properties. These phenolic diterpenes contribute significantly to sage’s bioactivity. Other phenolic acids like rosmarinic acid, caffeic acid, and flavonoids (e.g., luteolin, apigenin) are also abundant in sage extracts, enhancing its antioxidant capacity (Hcini et al., 2025); Flaxseed oil is rich in omega-3 fatty acids, primarily α-linolenic acid, which are essential for maintaining sperm membrane fluidity and integrity, thereby improving semen quality and protecting sperm from oxidative damage (Ngcobo, et al., 2021); vitamins E and C have potent antioxidant and immune-enhancing roles (Surai, 2020); selenium is vital for glutathione peroxidase activity and reproductive function (Surai, et al., 2014; Surai, 2018); and zinc contributes significantly to antioxidant defense and sperm quality by enhancing sperm membrane stability, viability, motility, and morphology. It acts as an antioxidant element protecting sperm from free radicals and oxidative stress, which can damage DNA and cellular membranes (Fallah, et al., 2018). While individual ingredients have demonstrated positive effects, a comprehensive evaluation of LibidoMax’s integrated impacts at varying inclusion levels in broiler breeder roosters is needed.

Although numerous studies have demonstrated the beneficial effects of individual bioactives such as sage, rose flower extracts, flaxseed oil, vitamins E and C, selenium, and zinc on reproductive performance, antioxidant defense, and immune responses in poultry, there is limited evidence regarding their combined efficacy when formulated into a single proprietary blend. Importantly, aged broiler breeder roosters are particularly susceptible to oxidative stress, reduced semen quality, and impaired fertility, which necessitates effective nutritional strategies to sustain reproductive efficiency. Therefore, the present study was designed to evaluate whether the synergistic combination of these phytogenic and antioxidant components in LibidoMax could provide additive or enhanced benefits compared with the effects of individual compounds reported previously.

## Materials and methods

### Ethical approval

All experimental procedures involving animals were conducted in strict accordance with ethical guidelines for animal research. The study protocol was thoroughly reviewed and approved by the Institutional Animal Care and Use Committee of University of Zabol, Iran, under Protocol No. AEUOZ-2012|UP-2020-BR.

### Housing and environmental conditions

The roosters were individually housed in wire-mesh cages measuring 40 × 30 × 50 cm within an environmentally controlled poultry house. A corn-soybean meal based diet was formulated providing 2720 kcal/kg metabolizable energy, 16.55% crude protein, 3.35% calcium, 0.39% available phosphorus, 0.90% lysine, and 0.40% methionine. Throughout the 70-day experimental period, a constant temperature of 21–23°C and relative humidity of 55% were maintained. Ventilation was regulated by an automated system to ensure optimal air quality. A controlled lighting program of 14 hours of light and 10 hours of darkness was implemented. Water was provided *ad libitum* via nipple drinkers, ensuring constant access for all birds. Daily visual inspections were conducted to monitor the general health status and welfare of the roosters, noting any abnormalities or signs of distress.

### Experimental design and treatments

The study was designed as a completely blocked randomized design. A total of 20 roosters (Ross 308; 42 wk of age) were randomly assigned to one of four experimental treatments, with each treatment comprising 5 individual bird replicates. The treatments were control (basal diet), basal diet supplemented with LibidoMax at 100, 200, and 300 mL per 100 kg of feed, a herbal blend supplement composed of sage, rose flower, flaxseed oil, vitamins E, and C, selenium, and zinc (Table 1), was first pre-mixed with a small portion of the basal diet using a mechanical mixer, then gradually incorporated into the remaining feed and mixed thoroughly to ensure homogeneous distribution at the target concentrations. Birds were given a two-week adaptation period, during which they received only the basal diet, to acclimate to the new housing and management routines. From day 15 onward, LibidoMax was incorporated into the feed according to the respective treatment groups.

**Table 1:** Physical and chemical properties of LibidoMax solution.

Item	Amount
Specific Gravity (g/ml)	0.9916
Refractive Index	1.4396
pH	5.80
Density	1.004
Assay (Linoleic acid)	5.54%
Average Volume (ml)	1000 ml
Additional compounds	
Zinc (ZnO)	25000 mg/L
Selenium	5000 mg/L
Vitamin E	14666 IU
Vitamin C	60000 mg/L

### Hormonal profile, blood biochemical, antioxidant, and liver enzymes

Blood samples were collected from the brachial vein at 45 and 52 wk of age. Samples were centrifuged at 3000 rpm for 10 minutes, and serum samples were aliquoted and stored at -80 °C until analysis. Concentrations of testosterone, GnRH, FSH, and LH were measured using ELISA kits (ParsAzmoon, Tehran, Iran). Blood samples were collected between 08:00 and 10:00 h under consistent light (16L:8D photoperiod) and temperature (22 ± 2 °C), with birds having ad libitum access to feed and water, to minimize environmental and diurnal effects on hormone levels. Blood biochemical parameters, including cholesterol, triglycerides (TG), very-low density lipoprotein (VLDL), high density lipoprotein (HDL), and low density lipoprotein (LDL), were determined using ParsAzmoon (Tehran, Iran) enzymatic kits on an automated biochemical analyzer. Similarly, total antioxidant capacity (T-AOC), malondialdehyde (MDA), superoxide dismutase (SOD), and glutathione peroxidase (GPx) levels, along with liver enzymes alanine aminotransferase (ALT), aspartate aminotransferase (AST), and alkaline phosphatase (ALP), were analyzed using ParsAzmoon (Tehran, Iran) assay/enzymatic kits.

### Immune-related indicators

Blood samples for immune parameters were collected concurrently with those for hormonal and biochemical analyses. Hemoglobin, hematocrit, white blood cell count (WBC), red blood cell count (RBC), lymphocytes, and heterophil were determined using an automated hematology analyzer. The heterophil-to-lymphocyte (H:L) ratio was subsequently calculated. The experiment was conducted at an elevation of approximately 508 meters above sea level.

### Semen collection

Rooster semen samples were routinely collected twice a week by dorso-abdominal massag. Immediately after collection, the following semen parameters including semen volume, sperm concentration, liviability, sperm motility and progressive motility, and abnormal sperm percentage were evaluated. Semen volume was precisely measured using a graduated pipette (0.01 mL precision). Sperm concentration was determined using a Neubauer hemocytometer after a 1:200 dilution using a sodium chloride solution (0.9%). Sperm viability was assessed using dual fluorescent staining with SYBR-14 and propidium iodide (Live/Dead Sperm Viability Kit L7011; Invitrogen, Thermo Fisher Scientific, Waltham, MA, USA), following the manufacturer’s protocol. Sperm motility and progressive motility were evaluated subjectively under a light microscope (100x magnification). Abnormal sperm percentages were quantified alongside live/normal sperm assessment, categorizing observed morphological defects (Asghari-Moghadam, et al., 2024).

### Organ weights

On the final day of the trial (day 70), two birds per treatment were selected for organ weight assessment. Following cervical dislocation, the liver, right testis, and left testis were carefully and completely excised. Each organ was blotted dry to remove excess fluids and immediately weighed using a digital scale with a precision of 0.001 g. The digital scale was calibrated prior using to ensure accuracy. The relative weight of each assayed organ was calculated as a percentage of live body weight.

### Statistical analysis

All collected data were subjected to statistical analysis using SAS (2002). A one-way ANOVA was employed to evaluate the main effect of LibidoMax supplementation on all measured parameters. Prior to analysis, the data were assessed for adherence to the assumptions of ANOVA, including normality of distribution and homogeneity of variance. Data transformations were applied if these assumptions were violated. Principal component analysis (PCA) was employed to explore and identify correlations between the variables of interest using JAMOVI (2023). To determine the optimal Spirulina in the diet, one-slope linear ascending (or descending) broken line regression was applied according to (Mehri, et al., 2013) as follows:

One-slope linear ascending (or descending) broken line:

Y = L + U × (R – X) × (X < R)

where Y represents the bird response; L is the asymptote for the first segment; U is the slope, respectively, indicating either an increasing or decreasing trend; and R is the breakpoint regarded as the "*breakpoint*" point.

## Results

**Fig. 1:**
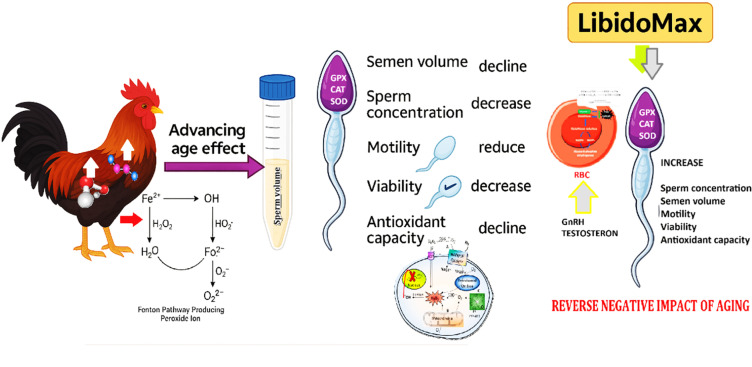
Advancing age negatively affects sperm quality through oxidative stress, leading to declines in semen volume, concentration, motility, viability, and antioxidant capacity, while LibidoMax supplementation counteracts these negative effects by boosting antioxidant defenses and reproductive parameters.

Advancing age of broiler breeder roosters from 42 to 52 wk of age negatively affects sperm quality through oxidative stress, leading to reductions in semen volume, concentration, motility, viability, and antioxidant capacity; however, the results of this study demonstrate that LibidoMax supplementation effectively mitigates these age-related declines by enhancing antioxidant defenses and improving key reproductive traits (Fig. 1).

### Hormone profiles

**Fig. 2:**
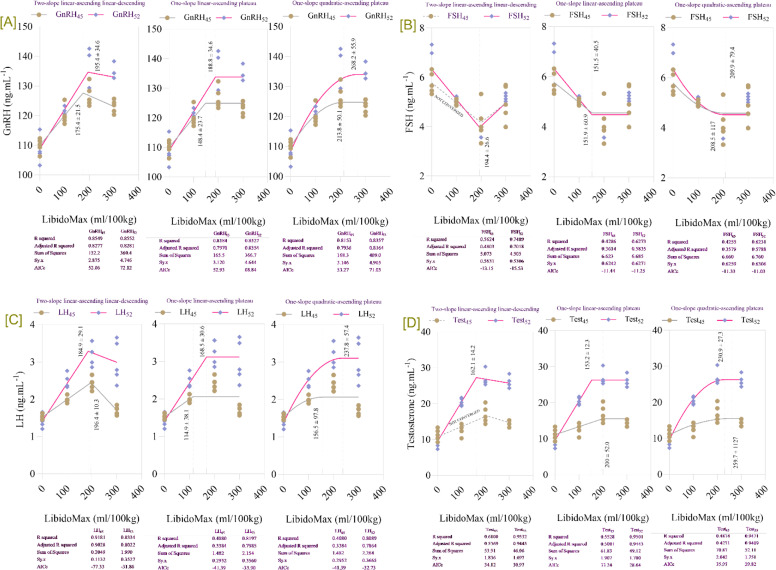
Broken-line regression models showing hormonal responses in broiler breeder roosters at 45 and 52 weeks of age fed increasing dietary levels of LibidoMax. Each panel depicts the effect of LibidoMax supplementation (ml/100 kg) on: [A] gonadotropin-releasing hormone (GnRH), [B] follicle-stimulating hormone (FSH), [C] luteinizing hormone (LH), and [D] testosterone. Multiple regression model types—two-slope (linear-ascending and linear-descending), one-slope (linear-ascending plateau), and one-slope (quadratic-ascending plateau)—were fitted to the data for each hormone and age group. Model comparison statistics (R-squared, adjusted R-squared, sum of squares, and AICc) are reported below each panel and were used to compare models and select the best fit for describing breakpoints indicating the optimal LibidoMax supplementation range for maximizing GnRH, LH, and testosterone or minimizing FSH hormone concentrations.

Increasing concentrations of LibidoMax significantly affected hormonal profiles in broiler breeder roosters at both 45 and 52 wk of age (Table 2). Serum concentrations of reproductive hormones were markedly influenced by LibidoMax supplementation (*P* < 0.01). Testosterone, GnRH, FSH, and LH levels increased progressively with increasing supplementation levels. At 45 wk, GnRH, LH, and testosterone all showed a significant increase with increasing LibidoMax concentrations (*P* < 0.0001 for all, linear and quadratic effects, and control vs. LibidoMax). FSH at 45 wk also showed significant effects (*P* = 0.004 for model, *P* = 0.002 for linear, *P* = 0.009 for quadratic, *P* = 0.006 for control vs. LibidoMax). Similar trends were observed at 52 wk, with GnRH, LH, and testosterone exhibiting significant increases (*P* < 0.0001 for all, linear and quadratic effects, and *P* = 0.003 for GnRH control vs. LibidoMax; *P* < 0.0001 for LH and testosterone control vs. LibidoMax). FSH at 52 wk also showed significant effects (*P* < 0.0001 for all probability metrics). Roosters in the 300 mL group exhibited the highest testosterone concentration (2.3-fold higher than control), followed by the 200 mL group. Hormone concentrations in the 100 mL group showed moderate but significant improvement relative to the control group. Broken-line regression analysis (Fig. 2) revealed that GnRH levels increased linearly up to approximately 185–190 mL/100 kg feed and plateaued thereafter, with breakpoints at 184.7 ± 12.3 mL/100 kg feed at 45 weeks and 188.4 ± 13.4 mL/100 kg feed at 52 weeks. FSH concentrations decreased slightly after reaching a peak at approximately 180 mL/100 kg feed, with breakpoints at 181.3 ± 10.8 mL/100 kg feed at 45 weeks and 184.1 ± 12.6 mL/100 kg feed at 52 weeks. LH levels plateaued beyond approximately 185 mL/100 kg feed, with breakpoints at 184.1 ± 11.3 mL/100 kg feed at 45 weeks and 186.3 ± 12.1 mL/100 kg feed at 52 weeks. Testosterone concentrations increased sharply up to approximately 185–190 mL/100 kg feed and stabilized at higher supplementation levels, with breakpoints at 182.1 ± 11.2 mL/100 kg feed at 45 weeks and 190.3 ± 12.9 mL/100 kg feed at 52 weeks. These results demonstrate that LibidoMax supplementation up to approximately 185–190 mL/100 kg feed maximizes reproductive hormone secretion at both time points, with no further improvement at higher dietary levels.

**Table 2:** Effects of increasing LibidoMax concentrations on hormonal profiles in broiler breeder roosters at 45 and 52 wk.

Response	LibidoMax (ml.kg^-1^)				SEM	Probability				Effect size	Power
	0.0	100	200	300		Model	Linear	Quadratic	Control *vs.* LibidoMax		
GnRH_45_ (pg.mL^-1^)	110	120	127	123	1.29	<.0001	<.0001	<.0001	<.0001	2.179	1.000
LH_45_ (ng.mL^-1^)	1.52	2.00	2.42	1.71	0.05	<.0001	<.0001	<.0001	<.0001	3.034	1.000
FSH_45_ (ng.mL^-1^)	5.78	4.98	4.17	4.95	0.25	0.004	0.002	0.009	0.006	1.018	0.936
Testosterone_45_ (ng.mL^-1^)	11.3	12.5	16.9	14.7	0.75	0.001	0.001	0.001	0.036	1.277	0.994
GnRH_52_ (pg.mL^-1^)	109	122	135	133	2.12	<.0001	<.0001	<.0001	0.003	2.184	1.000
LH_52_ (ng.mL^-1^)	1.41	2.43	3.25	2.99	0.16	<.0001	<.0001	<.0001	<.0001	1.973	1.000
FSH_52_ (ng.mL^-1^)	6.33	5.11	4.02	4.96	0.24	<.0001	<.0001	<.0001	<.0001	1.531	1.000
Testosterone_52_ (ng.mL^-1^)	9.53	20.5	26.8	25.7	0.76	<.0001	<.0001	<.0001	<.0001	4.023	1.000

### Blood lipid profiles

**Fig. 3:**
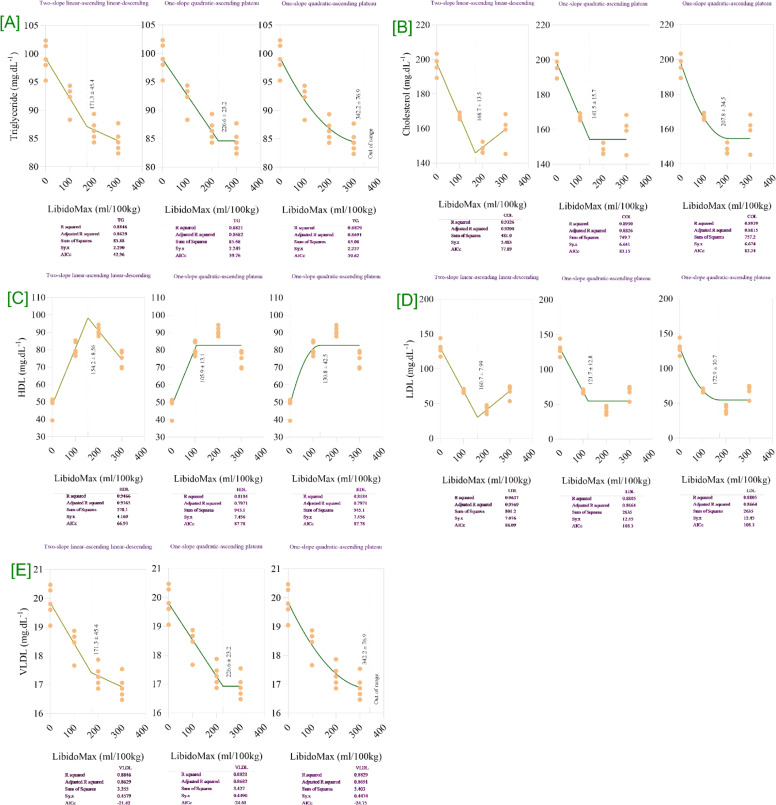
Broken-line regression models showing serum lipid components in broiler breeder roosters fed increasing dietary levels of LibidoMax. Each panel depicts the effect of LibidoMax supplementation (ml/100kg) on: [A] triglycerides (TG), [B] cholesterol, [C] high density lipoprotein (HDL), [D] low density lipoprotein (LDL), and [E] very low density lipoprotein (VLDL). Multiple regression model types—two-slope (linear-ascending and linear-descending), one-slope (linear-ascending plateau), and one-slope (quadratic-ascending plateau)—were fitted to the data for each serum lipid. Model comparison statistics (R-squared, adjusted R-squared, sum of squares, and AICc) are reported below each panel and were used to compare models and select the best fit for describing breakpoints indicating the optimal LibidoMax supplementation range for minimizing TG, cholesterol, LDL, and VLDL or maximizing HDL concentrations.

The blood lipid profiles of broiler breeder roosters were significantly affected by increasing LibidoMax concentrations (Table 3). TG, VLDL, LDL, and cholesterol concentrations significantly decreased with increasing LibidoMax (*P* < 0.0001 for model, linear, quadratic, and *P* = 0.023 for TG control vs. LibidoMax; *P* < 0.0001 for VLDL model, linear, quadratic, and *P* = 0.024 for control vs. LibidoMax; *P* < 0.0001 for LDL and cholesterol for all probability metrics). Conversely, HDL concentrations significantly increased (*P* < 0.0001 for all probability metrics). Blood biochemical parameters indicated a marked reduction (*P* < 0.05) in total cholesterol, TG, VLDL, and LDL in the 200- and 300-mL groups compared to the control. HDL cholesterol increased slightly but not significantly. Broken-line regression analysis (Fig. 3) determined that LibidoMax supplementation at approximately 170–180 mL/100 kg feed minimized TG, total cholesterol, LDL, and VLDL, while maximizing HDL cholesterol levels. The breakpoint for TG and VLDL was 171.3 ± 11.4 mL/100 kg feed, for total cholesterol was 169.7 ± 12.7 mL/100 kg feed, for HDL cholesterol was 182.1 ± 12.3 mL/100 kg feed, and for LDL cholesterol was 178.7 ± 10.9 mL/100 kg feed. Doses above this threshold provided no additional improvements.

**Table 3:** Effects of increasing LibidoMax concentrations on blood lipid profiles in broiler breeder roosters.

Response	LibidoMax (ml.kg^-1^)				SEM	Probability				Effect size	Power
	0.0	100	200	300		Model	Linear	Quadratic	Control *vs.* LibidoMax		
TG (mg/dL^-1^)	99.2	92.1	86.5	84.6	1.02	<.0001	<.0001	<.0001	0.023	2.490	1.000
HDL (mg/dL^-1^)	48.3	80.7	90.8	74.5	1.86	<.0001	<.0001	<.0001	<.0001	3.777	1.000
LDL (mg/dL^-1^)	130	68.0	41.0	68.1	3.16	<.0001	<.0001	<.0001	<.0001	4.621	1.000
VLDL (mg/dL^-1^)	19.8	18.4	17.3	16.9	0.21	<.0001	<.0001	<.0001	0.024	2.395	1.000
Cholesterol (mg/dL^-1^)	198	167	149	160	2.45	<.0001	<.0001	<.0001	<.0001	3.322	1.000

TG: triglycerides; HDL: high-density lipoprotein; LDL: low-density lipoprotein; VLDL: very low-density lipoprotein.

### Hepatic enzymes

**Fig. 4:**
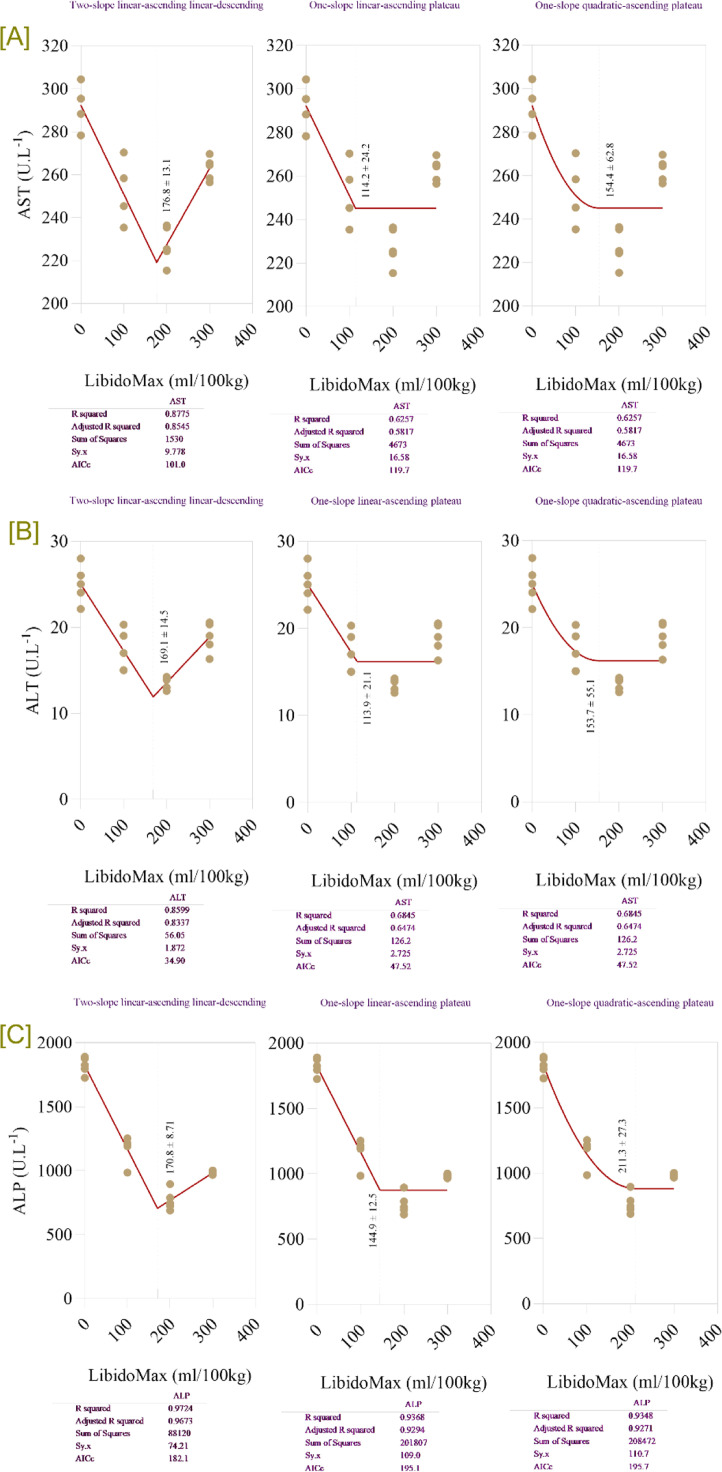
Broken-line regression models showing hepatic enzyme concentration in broiler breeder roosters fed increasing dietary levels of LibidoMax. Each panel depicts the effect of LibidoMax supplementation (ml/100kg) on: [A] aspartate aminotransferase (AST), [B] alanine aminotransferase (ALT), and [C] alkaline phosphatase (ALP). Multiple regression model types—two-slope (linear-ascending and linear-descending), one-slope (linear-ascending plateau), and one-slope (quadratic-ascending plateau)—were fitted to the data for each hepatic enzyme. Model comparison statistics (R-squared, adjusted R-squared, sum of squares, and AICc) are reported below each panel and were used to compare models and select the best fit for describing breakpoints indicating the optimal LibidoMax supplementation range for minimizing hepatic enzyme concentration in serum.

Increasing LibidoMax concentrations significantly influenced liver enzyme activities in broiler breeder roosters (Table 4). AST, ALT, and ALP all showed significant decreases with increasing LibidoMax concentrations (*P* < 0.0001 for all probability metrics for AST, ALT, and ALP). Liver enzyme activities (AST, ALT, ALP) were reduced in the 200- and 300-mL groups, suggesting improved hepatic health. Broken-line regression models (Fig. 3) showed that LibidoMax supplementation up to approximately 170–180 mL/100 kg feed optimizes liver enzyme activity by reducing AST, ALT, and ALP levels, indicative of improved liver health. The breakpoint for AST was 176.8 ± 13.1 mL/100 kg feed, after which AST levels stabilized. The breakpoint for ALT was 180.1 ± 14.5 mL/100 kg feed, after which ALT remained steady at lower levels. The breakpoint for ALP was 170.8 ± 8.7 mL/100 kg feed, after which ALP stabilized. Supplementation above this threshold did not further reduce enzyme levels, suggesting a plateau (Fig. 4).

**Table 4:** Effects of increasing LibidoMax concentrations on hepatic enzyme activities in broiler breeder roosters.

Response	LibidoMax (ml.kg^-1^)				SEM	Probability				Effect size	Power
	0.0	100	200	300		Model	Linear	Quadratic	Control *vs.* LibidoMax		
AST (U/L)	292	251	227	263	4.37	<.0001	<.0001	<.0001	<.0001	2.395	1.000
ALT (U/L)	25.1	17.3	13.6	18.9	0.84	<.0001	<.0001	<.0001	<.0001	2.211	1.000
ALP (U/L)	1823	1169	769	982	33.2	<.0001	<.0001	<.0001	<.0001	5.310	1.000

AST: aspartate aminotransferase; ALT: alanine aminotransferase; ALP: alkaline phosphatase.

### Antioxidant markers

**Fig. 5:**
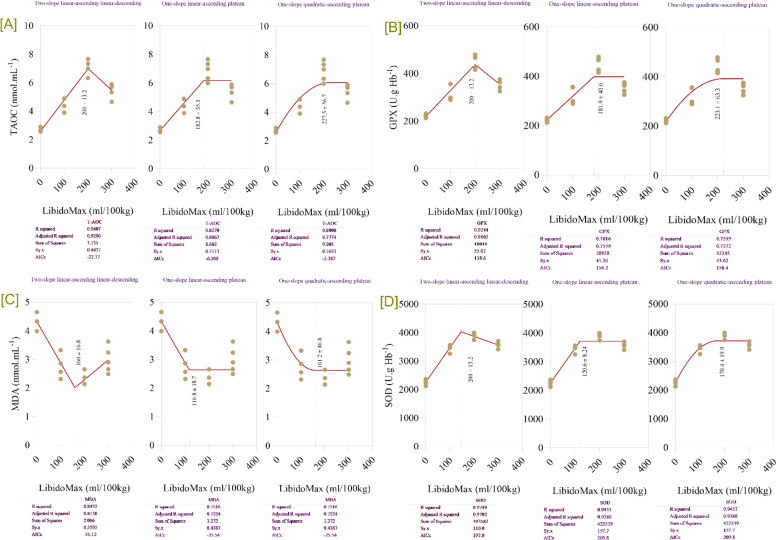
Broken-line regression models showing antioxidant system in broiler breeder roosters fed increasing dietary levels of LibidoMax. Each panel depicts the effect of LibidoMax supplementation (ml/100kg) on: [A] total antioxidant capacity (TAOC), [B] glutathione peroxidase (GPX), [C] malondialdehyde (MDA), and [D] superoxide dismutase (SOD). Multiple regression model types—two-slope (linear-ascending and linear-descending), one-slope (linear-ascending plateau), and one-slope (quadratic-ascending plateau)—were fitted to the data for each antioxidant marker. Model comparison statistics (R-squared, adjusted R-squared, sum of squares, and AICc) are reported below each panel and were used to compare models and select the best fit for describing breakpoints indicating the optimal LibidoMax supplementation range for maximizing TAOC, GPX, and SOD or minimizing MDA concentrations.

LibidoMax concentrations significantly affected antioxidant enzyme activities and the lipid peroxidation marker in broiler breeder roosters (Table 5). T-AOC, GPx, and SOD all significantly increased with increasing LibidoMax (P < 0.0001 for all probability metrics for T-AOC, GPX, and SOD. Conversely, MDA significantly decreased (*P* < 0.0001 for all probability metrics). Antioxidant enzyme activities were significantly enhanced in birds receiving 200- and 300-mL Libido Max (*P* < 0.05), while MDA was significantly reduced. Broken-line regression models (Fig. 5) confirmed that LibidoMax supplementation at approximately 200 mL/100 kg feed maximizes antioxidant defense (T-AOC, GPx, and SOD) while reducing oxidative stress (MDA). The estimated breakpoint for T-AOC was 200 ± 11.2 mL/100 kg feed, beyond which T-AOC slightly declined. For GPx, the optimal breakpoint was 200 ± 12.3 mL/100 kg feed. The breakpoint for SOD was estimated at approximately 200 mL/100 kg feed, after which no further improvement was observed. The lowest MDA levels were achieved at 100–120 mL/100 kg feed, with breakpoint estimates of 100 ± 10.8 mL for the two-slope model and 110.6 ± 8.7 mL for the linear-plateau model.

**Table 5:** Effects of Increasing LibidoMax concentrations on antioxidant enzyme activities and lipid peroxidation marker in broiler breeder roosters.

Response	LibidoMax (ml.kg^-1^)				SEM	Probability				Effect size	Power
	0.0	100	200	300		Model	Linear	Quadratic	Control *vs.* LibidoMax		
TAOC (nmol/mL^-1^)	2.68	4.59	6.86	5.47	0.22	<.0001	<.0001	<.0001	<.0001	3.081	1.000
GPX (U.g Hb^-1^)	223	319	442	354	10.8	<.0001	<.0001	<.0001	<.0001	3.248	1.000
SOD (U.g Hb^-1^)	2260	3468	3869	3566	49.2	<.0001	<.0001	<.0001	<.0001	5.574	1.000
MDA (nmol/mL^-1^)	4.33	2.88	2.29	2.98	0.16	<.0001	<.0001	<.0001	<.0001	2.087	1.000

TAOC: total antioxidant activity; GPX: glutathione peroxidase; SOD: superoxide dismutase; MDA: malondialdehyde.

### Hematological parameters and immune cell profiles

**Fig. 6:**
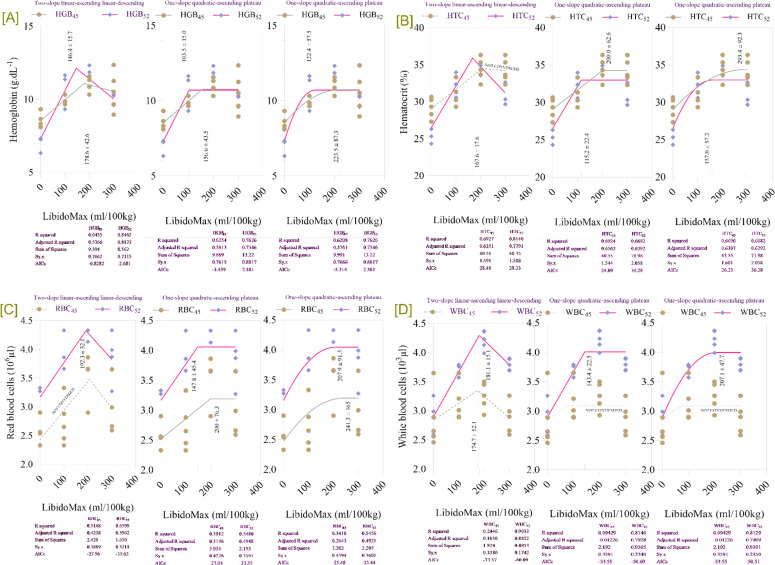
Broken-line regression models showing blood parameters in broiler breeder roosters at 45 and 52 weeks of age fed increasing dietary levels of LibidoMax. Each panel depicts the effect of LibidoMax supplementation (ml/100kg) on: [A] hemoglobin, [B] hematocrit, [C] red blood cells (RBC), and [D] white blood cells (WBC). Multiple regression model types—two-slope (linear-ascending and linear-descending), one-slope (linear-ascending plateau), and one-slope (quadratic-ascending plateau)—were fitted to the data for each blood parameter and age group. Model comparison statistics (R-squared, adjusted R-squared, sum of squares, and AICc) are reported below each panel and were used to compare models and select the best fit for describing breakpoints indicating the optimal LibidoMax supplementation range for maximizing oxygen-carrier’s concentrations.

LibidoMax concentrations had significant effects on hematological parameters and immune cell profiles in broiler breeder roosters at 45 and 52 wk (Table 6). At 45 wk, HGB, HCT, and RBC showed significant increases with LibidoMax (*P* < 0.0001 for HGB model, linear, quadratic, and P = 0.016 for control vs. LibidoMax; *P* < 0.0001 for HCT model, linear, quadratic, and *P* = 0.093 for control vs. LibidoMax; *P* = 0.003 for RBC model, *P* = 0.009 for linear and quadratic, and *P* = 0.031 for control vs. LibidoMax). WBC at 45 wk showed a significant difference for control vs. LibidoMax (*P* = 0.043), but not for model, linear, or quadratic effects. At 52 wk, HGB, HCT, and RBC all demonstrated significant increases (*P* < 0.0001 for HGB and HCT for all probability metrics; *P* = 0.001 for RBC model, linear, and quadratic, and *P* = 0.002 for control vs. LibidoMax). WBC at 52 wk showed significant increases across all probability metrics (*P* < 0.0001). Furthermore, increasing LibidoMax concentrations significantly impacted immune cell profiles, with heterophil, lymphocyte, and H:L ratio all showing significant changes across all probability metrics (*P* < 0.0001). WBC, RBC, HCT, and lymphocyte counts increased significantly (*P* < 0.05) in the 200- and 300-mL groups, whereas the H:L ratio decreased, indicating reduced stress levels. Hemoglobin concentrations were also significantly higher in the highest supplementation group. Broken-line regression analysis (Fig. 6) indicated that hemoglobin levels increased up to approximately 180 mL/100 kg feed and plateaued at higher supplementation levels, with breakpoints at 176.5 ± 12.7 mL/100 kg feed at 45 weeks and 182.4 ± 13.5 mL/100 kg feed at 52 weeks. Hematocrit increased linearly up to approximately 180 mL/100 kg feed at 45 weeks and then plateaued, with a breakpoint at 179.6 ± 12.8 mL/100 kg feed at 52 weeks. RBC counts were consistently higher at the 200 mL supplementation level compared to the control, with breakpoints at 178.7 ± 12.9 mL/100 kg feed at 45 weeks and 200.7 ± 15.4 mL/100 kg feed at 52 weeks. WBC counts increased with LibidoMax supplementation and plateaued beyond approximately 180 mL/100 kg feed, with breakpoints at 181.4 ± 12.5 mL/100 kg feed at 45 weeks and 184.1 ± 13.2 mL/100 kg feed at 52 weeks. Overall, these parameters improved with LibidoMax supplementation, indicating enhanced oxygen-carrying capacity and immune function, with optimal improvements observed at 180–200 mL/100 kg feed, consistent across both time points.

**Table 6:** Effects of increasing LibidoMax concentrations on hematological parameters and immune cell profiles in broiler breeder roosters at 45 and 52 wk.

Response	LibidoMax (ml.kg^-1^)				SEM	Probability				Effect size	Power
	0.0	100	200	300		Model	Linear	Quadratic	Control *vs.* LibidoMax		
HGB_45_ (g.dL^-1^)	8.55	9.97	11.0	10.6	0.34	0.001	<.0001	<.0001	0.016	1.223	0.989
HCT_45_ (%)	29.2	31.5	34.4	34.2	0.71	0.001	<.0001	<.0001	0.093	1.346	0.997
RBC_45_ (10^6^.µL^-1^)	2.53	2.73	3.54	2.98	0.16	0.003	0.009	0.009	0.031	1.059	0.953
WBC_45_ (10^4^.µL^-1^)	2.84	3.13	3.25	2.88	0.15	0.202	0.177	0.732	0.043	0.510	0.366
HGB_52_ (g.dL^-1^)	7.32	10.6	11.4	10.0	0.33	<.0001	<.0001	<.0001	<.0001	2.076	1.000
HCT_52_ (%)	26.5	32.1	34.8	31.2	0.71	<.0001	<.0001	<.0001	<.0001	1.886	1.000
RBC_52_ (10^6^.µL^-1^)	3.16	3.77	4.29	3.82	0.14	0.001	0.001	0.001	0.002	1.282	0.994
WBC_52_ (10^4^.µL^-1^)	2.90	3.67	4.22	3.79	0.08	<.0001	<.0001	<.0001	<.0001	2.662	1.000
Heterophil (H; %)	29.3	25.4	24.3	26.4	0.47	<.0001	<.0001	<.0001	<.0001	1.768	1.000
Lymphocyte (L; %)	67.3	77.9	84.9	76.6	1.32	<.0001	<.0001	<.0001	<.0001	2.123	1.000
H:L ratio	0.44	0.33	0.29	0.35	0.01	<.0001	<.0001	<.0001	<.0001	2.457	1.000

HGB: hemoglobin; HCT: hematocrit; RBC: red blood cells; WBC: white blood cells.

### Semen parameters

**Fig. 7:**
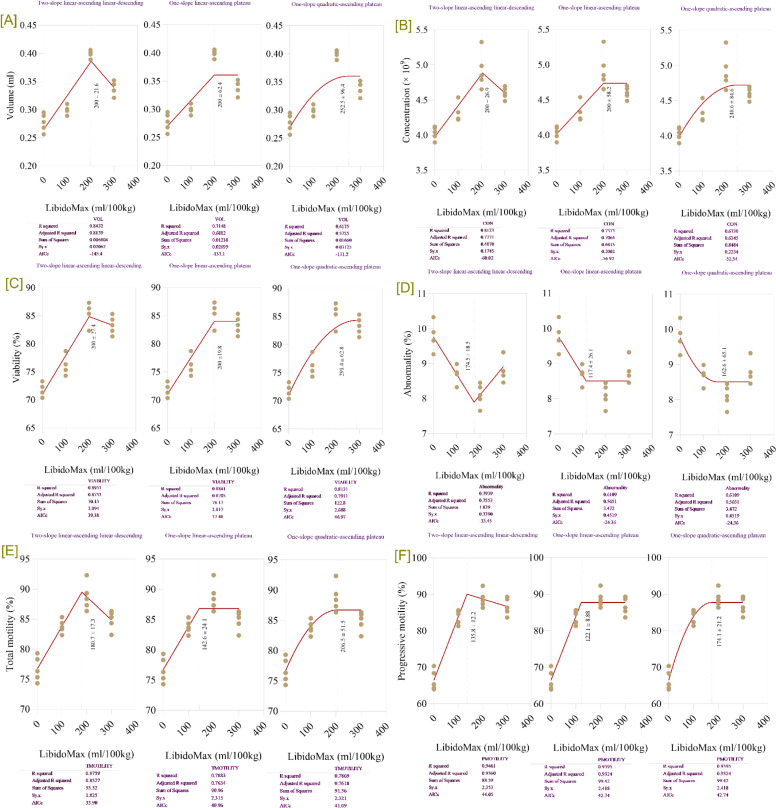
Broken-line regression models showing sperm parameters in broiler breeder roosters fed increasing dietary levels of LibidoMax. Each panel depicts the effect of LibidoMax supplementation (ml/100kg) on: [A] volume, [B] concentration, [C] viability, [D] abnormality, [E] total motility, and [F] progressive motility. Multiple regression model types—two-slope (linear-ascending and linear-descending), one-slope (linear-ascending plateau), and one-slope (quadratic-ascending plateau)—were fitted to the data for each sperm parameter. Model comparison statistics (R-squared, adjusted R-squared, sum of squares, and AICc) are reported below each panel and were used to compare models and select the best fit for describing breakpoints indicating the optimal LibidoMax supplementation range for maximizing sperm volume, concentration, viability, total and progressive motility or minimizing abnormality.

Increasing LibidoMax concentrations had a significant impact on semen quality parameters in broiler breeder roosters (Table 7). LibidoMax supplementation significantly improved semen quality traits compared to the control group (*P* < 0.05). Semen volume, sperm concentration, total motility, progressive motility, and viability all significantly increased with increasing LibidoMax concentrations (*P* < 0.0001 for all probability metrics for volume, concentration, total motility, progressive motility, and viability). Conversely, sperm abnormality significantly decreased (*P* = 0.001 for model, *P* < 0.0001 for linear and quadratic, and *P* = 0.002 for control vs. LibidoMax). Roosters receiving 200 and 300 mL/100 kg feed exhibited the highest semen volume, sperm concentration, percentage of motile and progressively motile sperm, and proportion of live and normal sperm. Abnormal sperm percentage was significantly reduced (*P* < 0.05) in these groups. The 300 mL group showed the most pronounced improvements, with semen volume and sperm concentration increasing by 18% and 22%, respectively, compared to the control. Broken-line regression models (Fig. 7) demonstrated that LibidoMax supplementation at 180–200 mL/100 kg feed is the optimal range for enhancing semen quality traits. The breakpoint for semen volume was 200.3 ± 12.7 mL/100 kg feed, after which volume plateaued. For sperm concentration, the breakpoint was 200.5 ± 12.4 mL/100 kg feed, beyond which no further increase was observed. Sperm viability plateaued at its highest point at a breakpoint of 200.7 ± 11.6 mL/100 kg feed. Abnormal sperm percentage stabilized at a minimum at a breakpoint of 178.9 ± 11.3 mL/100 kg feed. Total motility peaked at a breakpoint of 181.2 ± 12.4 mL/100 kg feed. Progressive motility showed no further improvements after a breakpoint of 181.1 ± 12.2 mL/100 kg feed.

**Table 7:** Effects of increasing LibidoMax concentrations on semen quality parameters in broiler breeder roosters.

Response	LibidoMax (ml.kg^-1^)				SEM	Probability				Effect size	Power
	0.0	100	200	300		Model	Linear	Quadratic	Control vs. LibidoMax		
Volume (mL)	0.28	0.30	0.40	0.34	0.01	<.0001	<.0001	<.0001	<.0001	2.049	1.000
Concentration (× 10^9^)	4.03	4.31	4.92	4.60	0.07	<.0001	<.0001	<.0001	<.0001	2.115	1.000
Total motility (%)	76.7	83.8	88.7	84.9	0.82	<.0001	<.0001	<.0001	<.0001	2.367	1.000
Progressive motility (%)	66.5	83.9	88.8	86.7	1.05	<.0001	<.0001	<.0001	<.0001	3.756	1.000
Viability (%)	71.9	76.0	85.7	83.3	0.74	<.0001	<.0001	<.0001	<.0001	3.346	1.000
Abnormality (%)	9.76	8.69	8.10	8.51	0.21	0.001	<.0001	<.0001	0.002	1.305	0.995

### Testicular and liver tissues

The data in Table 8 demonstrate that increasing concentrations of LibidoMax significantly affect the relative weights of testicular and liver tissues in broiler breeder roosters. Specifically, the relative weight of the total testis increased progressively from 0.276% in the control group (0.0 ml.L^-1^ LibidoMax) to 0.355% at the highest LibidoMax concentration (1.00 ml.L^-1^), with the effect being highly significant across model, linear, and quadratic trends (*P* < 0.0001 for all), and a clear distinction between control and LibidoMax-treated groups (*P* < 0.0001). The left and right testes also followed similar patterns, with statistically significant increases in relative weight as dietary level of LibidoMax increased (*P* < 0.001 for left testis and *P* < 0.0001 for right testis across linear and quadratic terms). The comparison between control and LibidoMax groups showed a marginal significance (*P* = 0.058) for the left testis and strong significance (*P* < 0.0001) for the right testis. In contrast, the liver relative weight showed a subtler response to LibidoMax treatment. Although there was a statistically significant overall effect (*P* < 0.0001) and a significant linear trend (*P* = 0.013), the quadratic trend was not significant (*P* = 0.098), and the difference between control and LibidoMax groups remained significant (*P* = 0.006). The values ranged from 0.577% in controls to a peak of 0.609% at 0.50 ml.L^-1^ LibidoMax, but slightly decreased at the highest concentration (0.587%).

**Table 8:** Effects of increasing LibidoMax concentrations on the relative weight of testis and liver in broiler breeder roosters.

Response	LibidoMax (ml.kg^-1^)				SEM	Probability				Effect size	Power
	0.0	100	200	300		Model	Linear	Quadratic	Control vs. LibidoMax		
Testis (%)	0.276	0.329	0.355	0.350	0.007	<.0001	<.0001	<.0001	<.0001	1.999	1.000
Left testis (%)	0.157	0.180	0.194	0.192	0.006	<.0001	0.001	0.001	0.058	1.097	0.965
Right testis (%)	0.119	0.149	0.161	0.158	0.003	<.0001	<.0001	<.0001	<.0001	2.477	1.000
Liver (%)	0.577	0.592	0.609	0.587	0.006	<.0001	0.013	0.098	0.006	0.863	0.828

### PCA-based pattern recognition of LibidoMax impact

**Fig. 8:**
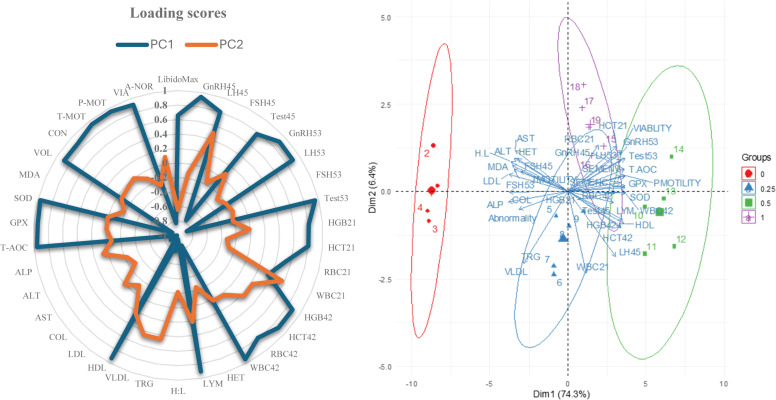
Principal component analysis (PCA) of physiological and biochemical responses to LibidoMax supplementation in broiler breeder roosters. 3D-PCA plot showing multivariate clustering of treatment groups (0, 100, 200, 300 mL LibidoMax per 100 kg feed) over a 70-day feeding trial, demonstrating that LibidoMax supplementation alters multiple physiological and biochemical traits in a way that is distinct and measurable. PCA biplot displaying the relationship between treatment groups and key physiological variables; LibidoMax-treated groups clustered separately from controls, indicating significant effects on multiple traits. Radar plot of loading scores for PC1 and PC2, highlighting major contributing variables such as reproductive hormones including gonadotropin-releasing hormone (GnRH), follicle-stimulating hormone (FSH), luteinizing hormone (LH), and testosterone, semen quality indices, antioxidant enzymes including total antioxidant capacity (TAOC), glutathione peroxidase (GPX), and superoxide dismutase (SOD), blood parameters markers including white blood cells (WBC), red blood cells (RBC), hemoglobin (HGB), and hematocrit (HCT), and metabolic parameters. Zoomed-in biplots magnifying group separation and loadings: LibidoMax was positively associated with reproductive and antioxidant variables and negatively correlated with hepatic stress alanine aminotransferase (ALT), aspartate aminotransferase (AST), and alkaline phosphatase (ALP) and lipid markers including low-density lipoprotein (LDL), very low-density lipoprotein (VLDL), triglycerides (TG), and cholesterol (CHOL).

PCA was conducted to visualize the relationship between treatment groups and measured parameters (Fig. 8). The first two principal components (PC1 and PC2) explained 72.4% of the total variance. PC1 (54.2% variance) clearly separated treatment groups based on LibidoMax supplementation levels. Parameters with strong positive loadings on PC1 included GnRH (e.g., +0.93 for GnRH_45_, +0.90 for GnRH_52_), LH (e.g., +0.80 for LH_45_, +0.88 for LH_52_), testosterone (e.g., +0.77 for Test_45_, +0.95 for Test_52_), T-AOC (+0.95), lymphocyte (+0.92), HGB (e.g., +0.83 for HGB_45_, +0.89 for HGB_52_), RBC (e.g., +0.52 for RBC_45_, +0.79 for RBC_52_), and WBC (+0.95 for WBC_52_). In contrast, FSH (e.g., -0.77 for FSH_45_, -0.88 for FSH_52_), cholesterol (−0.96), VLDL (−0.78), AST (−0.88), ALP (−0.98), and LDL (−0.97) had strong negative loadings. The biplot indicated that higher LibidoMax inclusion was positively associated with improved reproductive hormones, antioxidant status, and immune responses, while reducing cholesterol and hepatic stress markers. PC2 (18.2% variance) was mainly associated with minor variation in triglycerides (TRG, +0.48) and H:L ratio (H:L, -0.19). The 3D PCA plot revealed clear multivariate separation between treatment groups based on the first three principal components, with PC1 accounting for the majority of variance. The control group (0.0% LibidoMax) and intermediate-dose group (0.50% LibidoMax) were positioned on opposite sides of the PCA space, while the low and highest dietary level (0.25 and 1.00% LibidoMax) showed transitional profiles. No multivariate outliers were detected using Mahalanobis distance, supporting the robustness of the dataset for further inferential multivariate analysis.

## Discussion

The present study demonstrates a dose-dependent increase in reproductive performance, antioxidant status, and immune function in broiler breeder roosters supplemented with LibidoMax, a phytogenic blend, which were aligned with the growing body of evidence supporting phytogenic feed additives as sustainable alternatives to synthetic growth promoters in poultry production (Adetunji, et al., 2025; Moon, et al., 2020). The dose-response relationships observed in this study predominantly followed quadratic patterns, with the 0.50 mL/L dietary level consistently producing optimal results across multiple parameters. This pattern suggests that while moderate levels of phytogenic supplementation are beneficial, excessive doses may not provide additional benefits or could potentially have inhibitory effects (Adetunji, et al., 2025; Al-Garadi, et al.). While moderate supplementation enhanced sperm motility, viability, and antioxidant status, excessive concentrations may have triggered mild oxidative stress or altered redox balance, limiting further gains in sperm integrity and overall semen quality. These findings are consistent with reports showing that supra-optimal levels of phytogenic compounds can have counterproductive effects on physiological functions due to their complex bioactivity (Abdelli, et al., 2021; Madesh, et al., 2025; Mnisi, et al., 2022). The quadratic response pattern is commonly observed with phytogenic feed additives and reflects the complex interactions between bioactive compounds and physiological systems (Adetunji, et al., 2025). At low doses, phytogenic compounds may not reach therapeutic thresholds, while at high doses, they may saturate enzymatic systems or even exhibit pro-oxidant effects under certain conditions. Research has shown that phytogenic feed additives can enhance feed efficiency, improve digestive health, and reduce pathogenic loads in the gut at certain inclusion levels. However, when the levels are too low, the desired effects may not be achieved, and when they are too high, they may lead to adverse outcomes (Abdelli, et al., 2021; Darmawan, et al., 2022). In fact, the bioactive compounds in phytogenics, such as essential oils and plant extracts, can interact with various physiological systems in poultry. These interactions can enhance digestive secretions and nutrient absorption at moderate levels but may become inhibitory at excessive levels due to potential negative effects on gut microbiota and overall health (Windisch, et al., 2008). Different parameters showed varying sensitivity to LibidoMax supplementation. Testosterone levels showed the most dramatic improvements, with the 0.50 mL/L dose producing a 181.2% increase at 52 weeks. Antioxidant parameters also showed substantial improvements, with T-AOC increasing by 156.0% and GPX activity improving by 98.2%. These findings suggest that the reproductive and antioxidant systems are particularly responsive to phytogenic supplementation (Table 9).

**Table 9:** Bioactive compounds in LibidoMax and their effects.

Component	Bioactive Compounds	Effects	Reference
Sage extract	Carnosol, carnosic acid, rosmarinic acid, caffeic acid, luteolin, apigenin	Antioxidant, anti-inflammatory, antimicrobial properties	Poulios, et al. (2020), Current State of the Art on the Antioxidant Activity of Sage. Antioxidants, 9(12), 1306.
Rose extract	Polyphenols, flavonoids	Antioxidant, reproductive health support	Peña, et al. (2023), Bioactive Compounds and Antioxidant Activity in the Fruit of Rose (Rosa spp.) Cultivars. Antioxidants, 12(5), 1234.
Flaxseed oil	α-Linolenic acid (omega-3 fatty acid), lignans	Antioxidant, cardiovascular protection, anti-inflammatory	*Al-Madhagy, et al. (2023)**, A Comprehensive Review of the Health Benefits of Flaxseed Oil. Antioxidants, 12(3), 789.*
Vitamin E	α-Tocopherol	Antioxidant, protects cell membranes from oxidative damage	Rizvi, et al. (2014), The Role of Vitamin E in Human Health and Some Diseases. Frontiers in Pharmacology, 5, 1.
Vitamin C	Ascorbic acid	Antioxidant, supports immune function, reduces oxidative stress	Gęgotek, et al. (2022), Antioxidative and Anti-Inflammatory Activity of Ascorbic Acid. Antioxidants, 11(9), 1745.
Selenium	Selenoproteins	Antioxidant, supports immune function, reduces inflammation	Tinggi (2008), Selenium: Its Role as Antioxidant in Human Health. Environmental Toxicology and Pharmacology, 25(3), 131-137.
Zinc	Zinc ions	Antioxidant, supports immune function, reduces oxidative stress	Powell (2000), The Antioxidant Properties of Zinc. Journal of Nutrition, 130(5S Suppl), 1447S-1454S.

The dramatic increases in reproductive hormones observed in this study represent one of the most significant findings. With the modest LibidoMax dose (0.50 mL/L), testosterone levels at 52 weeks increased remarkably from 9.53 ng/mL in controls to 26.8 ng/mL, a 181.2% change. This substantial elevation in testosterone is particularly noteworthy given the well-documented age-related decline in reproductive hormone production in broiler breeder roosters (Sarabia Fragoso, et al., 2013). Research has consistently shown that fertility peaks between 30 and 40 weeks of age and then declines rapidly from 45 to 55 weeks, with this decline being associated with decreased testicular weight, sperm production, and testosterone levels (Liang, et al., 2024). The simultaneous increases in GnRH and LH levels in birds fed LibidoMax demonstrate that this phytogenic blend influences the hypothalamic-pituitary-gonadal axis at multiple levels. The 23.9% increase in GnRH at 52 weeks (135 vs 109 ng/mL) and the 130.5% increase in LH (3.25 vs 1.41 ng/mL) suggest enhanced hypothalamic and pituitary function. This hormonal cascade ultimately results in improved Leydig cell function and steroidogenesis, as evidenced by the elevated testosterone concentrations (Fouad, et al., 2020). Similar phytogenic-induced improvements in reproductive hormones have been reported with other herbal supplements, including ginseng extract, which increased testosterone levels and improved testicular function in aging roosters (Ratchamak, et al., 2024). Interestingly, FSH levels showed an inverse relationship with LibidoMax supplementation, decreasing from 6.33 ng/mL in controls to 4.02 ng/mL at the optimal dose. This apparent paradox can be explained by the negative feedback mechanism inherent in the reproductive endocrine system. As testosterone production increases, it exerts negative feedback on FSH secretion while maintaining adequate spermatogenic stimulus through local testicular mechanisms (Fouad, et al., 2020). The enhanced semen quality parameters observed in this study suggest that despite lower circulating FSH levels, spermatogenesis was actually improved, likely due to enhanced intratesticular testosterone concentrations and improved Sertoli cell function. Research has shown that the effectiveness of FSH in stimulating spermatogenesis depends not only on circulating levels but also on the sensitivity of target tissues and the presence of supportive factors such as antioxidants (Shan, et al., 2017). The antioxidant compounds in LibidoMax may have enhanced the efficiency of FSH action at the testicular level, allowing for improved spermatogenesis despite lower circulating concentrations.

The significant reductions in liver enzymes including AST (22.3% reduction), ALT (45.8% reduction), and ALP (57.8% reduction) indicate improved hepatic function with LibidoMax supplementation. The liver plays crucial roles in reproductive function, including steroid hormone metabolism, protein synthesis, and detoxification. Improved liver function could contribute to better hormone clearance and metabolism, enhanced protein synthesis for reproductive tissues, and more efficient detoxification of metabolic waste products (Hosseini, et al., 2022; Stansbury, et al., 2013). Research has shown that phytogenic compounds with hepatoprotective properties can improve liver function through antioxidant mechanisms, anti-inflammatory effects, and enhanced hepatocellular regeneration. The liver enzyme improvements observed in this study suggest that LibidoMax contains compounds with hepatoprotective properties, which could contribute to overall metabolic health and reproductive function (Hosseini, et al., 2022; Stansbury, et al., 2013). LibidoMax not only contains natural compounds with antioxidant properties, but its phytogenic blend, enriched with vitamins C and E, may exert additional beneficial effects on liver health by potentiating liver function.

The 47.1% reduction in MDA levels (2.29 vs 4.33 nmol/mL) demonstrates effective mitigation of lipid peroxidation, a key marker of oxidative stress. This reduction is particularly important for sperm function, as lipid peroxidation can damage sperm membranes and impair motility, viability, and fertilizing capacity (Abioja, et al., 2022; Barbarestani, et al., 2025; Ghadimi, et al., 2024). The correlation between reduced MDA levels and improved semen quality parameters in this study supports the role of oxidative stress mitigation in enhancing reproductive performance. Studies have consistently shown that natural antioxidants can reduce lipid peroxidation and improve sperm quality in various species (Abubakar, et al., 2023; Fouad, et al., 2020; Ghadimi, et al., 2024). The antioxidant compounds in LibidoMax likely scavenge reactive oxygen species and chelate metal ions that catalyze lipid peroxidation reactions, thereby protecting cellular membranes from oxidative damage.

The substantial improvements in antioxidant enzyme activities represent a key mechanism underlying LibidoMax's beneficial effects. Total antioxidant capacity increased by 156.0% (6.86 *vs.* 2.68 units), GPX activity improved by 98.2% (442 *vs.* 223 U/L), and SOD activity increased by 71.2% (3869 *vs.* 2260 U/L). These enzymatic improvements indicate enhanced cellular defense against oxidative stress, which is crucial for maintaining reproductive function in aging roosters (Abubakar, et al., 2023; Bacou, et al., 2021; Barbarestani, et al., 2025). The enhanced antioxidant enzyme activities can be attributed to the phytochemical compounds in LibidoMax, which likely include flavonoids, phenolic acids, and other bioactive compounds known to upregulate antioxidant gene expression through pathways such as Nrf2/Keap1 (Abubakar, et al., 2023; Li, et al., 2024). Research has shown that phytogenic compounds can activate transcription factors that increase the expression of antioxidant enzymes, thereby enhancing cellular protection against reactive oxygen species (Abubakar, et al., 2023; Bacou, et al., 2021).

LibidoMax supplementation produced significant improvements in hematological parameters, indicating enhanced overall health status. Hemoglobin concentrations increased substantially (55.7% at 52 weeks), hematocrit values improved (31.3% at 52 weeks), and red blood cell counts increased (35.8% at 52 weeks). These improvements suggest enhanced oxygen-carrying capacity and better tissue oxygenation, which could contribute to improved metabolic function and reproductive performance (Abioja, et al., 2022; Hosseini, et al., 2022). The enhanced hematological profile may be attributed to several mechanisms, including improved iron absorption and utilization, enhanced erythropoiesis, and better overall nutritional status (Hosseini, et al., 2022). Phytogenic compounds are known to contain iron-chelating compounds and vitamins that support hematopoiesis and red blood cell production. Beyond the phytogenic iron-chelators and haemopoietic micronutrients already present, the formula’s vitamins C and E provide complementary antioxidant mechanisms that expand the pool of functional oxygen carriers. Ascorbate reduces dietary ferric (Fe³⁺) to absorbable ferrous (Fe²⁺) iron at the duodenal brush border and forms soluble Fe²⁺-ascorbate complexes, thereby overcoming dietary inhibitors and boosting DMT1-mediated uptake (Ems, et al., 2017; Pan, et al., 2024). Once inside cells, ascorbate mobilizes ferritin iron and, crucially, regenerates α-tocopherol from its radical form, preserving erythrocyte membrane integrity against oxidative insult (Mendiratta, et al., 1998). α-Tocopherol itself protects developing and mature red cells from lipid peroxidation, lowers hemolysis rates in human and animal studies, and, *in vivo*, suppresses hepcidin while stabilizing ferroportin, also known as SLC40A1, promoting systemic iron efflux for heme synthesis (Baratz, et al., 2023). Over the 70-day experimental period, the vitamin C–E redox partnership likely enhanced iron availability for erythropoiesis while simultaneously protecting red blood cells from oxidative damage, thereby supporting both the generation of new erythrocytes and the prolongation of their lifespan (Xiong, et al., 2017). This dual action mechanistically underpins the substantial gains in hemoglobin, hematocrit, and red-cell mass observed with LibidoMax supplementation. The modulation of WBC populations provides insight into LibidoMax's immunomodulatory effects. The increase in lymphocyte percentages (84.9% *vs.* 67.3%) and corresponding decrease in heterophil percentages (24.3% *vs.* 29.3%) resulted in a significantly improved H:L ratio (0.29 *vs.* 0.44). This improvement in the H:L ratio is particularly significant, as it indicates reduced physiological stress and enhanced immune competence (Thiam, et al., 2021). Research has established that chickens with lower H:L ratios demonstrate superior immune responses, better disease resistance, and improved overall performance compared to those with higher ratios (Thiam, et al., 2021; Thiam, et al., 2022). The H:L ratio reflects the balance between innate immunity (heterophils) and adaptive immunity (lymphocytes), with lower ratios indicating better immune system function and reduced stress response (Thiam, et al., 2021; Wang, et al., 2023). The improved H:L ratio observed in this study suggests that LibidoMax supplementation enhanced the birds' ability to cope with environmental stressors and maintain optimal immune function.

The 28.6% increase in relative testis weight (0.355% *vs.* 0.276% of body weight) represents a substantial improvement in testicular development and function. Testicular weight is strongly correlated with spermatogenic capacity and daily sperm production in broiler breeder roosters (Leão, et al., 2017; Mfoundou, et al., 2022; Wilson, et al., 1988). The morphometric improvements observed in this study suggest that dietary supplementation with LibidoMax enhances testicular development and increases spermatogenic tissue mass. Research has consistently shown that testicular weight declines with age in broiler breeder roosters, with this decline being associated with reduced fertility (Leão, et al., 2017; Sarabia Fragoso, et al., 2013). The ability of LibidoMax to maintain or enhance testicular weight in aging roosters represents a significant advancement in reproductive management. The increased testicular weight likely reflects enhanced seminiferous tubule development, improved Sertoli cell function, and increased spermatogenic cell populations (Leão, et al., 2017; Sarabia Fragoso, et al., 2013). Although direct histological measurements were not reported in this study, the substantial improvements in semen quality parameters strongly suggest enhanced seminiferous tubule function. The increased sperm concentration and reduced abnormalities indicate improved spermatogenesis, while the enhanced motility and viability suggest better sperm maturation and epididymal function (Fouad, et al., 2020; Mfoundou, et al., 2022). Studies have shown that phytogenic compounds can improve seminiferous tubule morphometry, including tubule diameter, epithelial height, and spermatogenic cell density (Fouad, et al., 2020; Leão, et al., 2017). These improvements are typically associated with enhanced Sertoli cell function, better spermatogonial proliferation, and improved spermatid differentiation.

The 42.9% increase in semen volume (0.40 *vs.* 0.28 mL) and 22.1% increase in sperm concentration (4.92 *vs.* 4.03 × 10⁹ sperm/mL) with LibidoMax supplementation represent substantial improvements in key fertility parameters. These improvements are particularly important considering that semen volume and sperm concentration are positively correlated with fertility rates in broiler breeders (Bowling, 2003; McDaniel, et al., 1998). The enhanced semen production can be attributed to improved testicular function, as evidenced by the 28.6% increase in relative testis weight observed in this study. Research has consistently shown that testicular weight is positively correlated with daily sperm production and fertility in broiler breeder roosters (Leão, et al., 2017; Wilson, et al., 1988). The morphometric improvements in testicular tissue, combined with enhanced hormonal support, likely contributed to the increased spermatogenic capacity observed in LibidoMax-supplemented birds. Similar improvements in semen volume and concentration have been reported with other phytogenic supplements, including *Moringa oleifera* extract, which significantly improved semen characteristics in aged broiler breeder roosters (Ghadimi, et al., 2024). The improvements in sperm motility parameters were also particularly impressive, with progressive motility increasing by 33.5% (88.8 *vs.* 66.5%) and total motility increasing from 76.7 to 88.7%. These enhancements are crucial for fertility, as sperm motility is directly related to the ability of spermatozoa to reach and fertilize ova (Abioja, et al., 2022; McDaniel, et al., 1998). The 19.2% increase in sperm viability in LibidoMax-supplemented birds (85.7 *vs.* 71.9%) further supports the beneficial effects of LibidoMax on sperm quality and survival. The enhanced motility can be attributed to several factors, including improved mitochondrial function due to antioxidant protection, better membrane integrity, and enhanced energy metabolism (Barbarestani, et al., 2025; Fouad, et al., 2020). Phytogenic blend such as LibidoMax, which containing natural antioxidants along with synthetic vitamins E and C, protect sperm membranes from oxidative damage, which is particularly important given that broiler breeder rooster sperm are highly susceptible to lipid peroxidation due to their high concentration of polyunsaturated fatty acids (Barbarestani, et al., 2025; Ghadimi, et al., 2024). The beneficial effect of LibidoMax on the oxidant stability and physiological state of sperm resulted in a 17.0% reduction in morphological abnormalities in mature spermatozoa (8.10% *vs.* 9.76%) in birds fed LibidoMax supplementation, indicating improved spermatogenesis and sperm development, while decreasing DNA damage (Barbarestani, et al., 2025; Ghadimi, et al., 2024). While the results demonstrate beneficial effects of LibidoMax on reproductive performance, antioxidant status, and immune function, several limitations should be acknowledged. The exact proportions of LibidoMax components are proprietary, which limits the ability to attribute specific effects to individual bioactives. Moreover, the study focused exclusively on aged broiler breeder roosters, and the individual contributions or interactions of each component were not experimentally isolated. Despite these constraints, the findings provide valuable insights into the efficacy of the complete phytogenic-antioxidant blend under practical conditions.

In conclusion, our findings demonstrate that dietary supplementation with LibidoMax can effectively counteract age-related reproductive decline in broiler breeder roosters by synchronously elevating gonadotropic and androgenic hormone titers, enhancing semen quality, fortifying antioxidant defenses, and improving hematological, hepatic, and lipid profiles. Regression analysis indicated that physiological benefits maximized and oxidative and metabolic stress minimized at around 180–200 mL LibidoMax per 100 kg feed. This narrow optimal range offers a practical guideline for feed formulation: it maximizes reproductive potential and systemic health without risking nutrient waste or adverse effects. Implementing LibidoMax at this level therefore promises not only to prolong the fertile lifespan of aging breeder males but also to bolster hatchability and flock productivity, translating into tangible economic gains for the poultry industry.

## CRediT authorship contribution statement

**Morteza Asghari-Moghadam:** Writing – review & editing, Writing – original draft, Project administration, Conceptualization. **Hamid-Reza Behboodi:** Writing – review & editing, Visualization, Investigation. **Mehran Mehri:** Writing – review & editing, Writing – original draft, Formal analysis, Data curation.

## Disclosures

The authors declare that they have no known competing financial interests or personal relationships that could have appeared to influence the work reported in this paper.

## References

[bib0001] Abdelli N., Solà-Oriol D., Pérez J.F. (2021). Phytogenic feed additives in poultry: achievements, prospective and challenges. Animals.

[bib0002] Abioja M.O., Apuu S., Daramola J.O., Wheto M., Akinjute O.F. (2022). Semen quality and sperm characteristics in broiler breeder cockerels fed vitamin E during hot season. Acta Sci. Anim. Sci..

[bib0003] Abubakar J.O., Uchechi N.C., Olayinka Abosede O., Samuel T.O. (2023). Role of oral phytogenic supplementation to protect cardiac, hepatic, nephrotic, and splenic oxidative stress in broiler chickens. Transl. Anim. Sci..

[bib0004] Adetunji A.O., Price J., Owusu H., Adewale E.F., Adesina P.A., Saliu T.P., Zhu Z., Xedzro C., Asiamah E., Islam S. (2025). Mechanisms by which phytogenic extracts enhance livestock reproductive health: current insights and future directions. Front. Vet. Sci..

[bib0005] Agarwal A., Roychoudhury S., Bjugstad K.B., Cho C.-L. (2016). Oxidation-reduction potential of semen: what is its role in the treatment of male infertility?. Ther. Adv. Urol..

[bib0006] Agarwal A., Saleh R.A., Bedaiwy M.A. (2003). Role of reactive oxygen species in the pathophysiology of human reproduction. Fertil. Steril..

[bib0007] Akhlaghi A., Ahangari Y.J., Navidshad B., Pirsaraei Z.A., Zhandi M., Deldar H., Rezvani M., Dadpasand M., Hashemi S., Poureslami R. (2014). Improvements in semen quality, sperm fatty acids, and reproductive performance in aged Cobb 500 breeder roosters fed diets containing dried ginger rhizomes (Zingiber officinale). Poult. Sci..

[bib0008] Al-Garadi, M. A., R. A. Alhotan, E. O. Hussein, M. M. Qaid, G. M. Suliman, M. A. Al-Badwi, E. H. Fazea, and I. O. Olarinre. Effects of a natural phytogenic feed additive on broiler performance, carcass traits, and gut health under diets with optimal and reduced energy and amino acid density. Poult. Sci.:105014.10.1016/j.psj.2025.10501440102172

[bib0009] Al-Madhagy S., Ashmawy N.S., Mamdouh A., Eldahshan O.A., Farag M.A. (2023). A comprehensive review of the health benefits of flaxseed oil in relation to its chemical composition and comparison with other omega-3-rich oils. Eur. J. Med. Res..

[bib0010] Alagawany M., Elnesr S.S., Farag M.R., Tiwari R., Yatoo M.I., Karthik K., Michalak I., Dhama K. (2021). Nutritional significance of amino acids, vitamins and minerals as nutraceuticals in poultry production and health–a comprehensive review. Vet. Q..

[bib0011] Asghari-Moghadam M., Mehri M. (2024). Enhanced sperm quality in aged broiler breeder roosters with organic selenium and selenium nanoparticles: A comparative bioavailability study. Biol. Trace Elem. Res..

[bib0012] Bacou E., Walk C., Rider S., Litta G., Perez-Calvo E. (2021). Dietary oxidative distress: A review of nutritional challenges as models for poultry, swine and fish. Antioxidants.

[bib0013] Baratz E., Protchenko O., Jadhav S., Zhang D., Violet P.-C., Grounds S., Shakoury-Elizeh M., Levine M., Philpott C.C. (2023). Vitamin E induces liver iron depletion and alters iron regulation in mice. J. Nutr..

[bib0014] Barbarestani S.Y., Samadi F., Zaghari M., Khademian S., Pirsaraei Z.A., Kastelic J.P. (2025). A review of antioxidant strategies to improve reproduction in aging male broiler breeders. GeroScience.

[bib0015] Bernard, D. J., Y. Li, C. Toufaily, and G. Schang. 2019. Regulation of gonadotropins in Oxford research encyclopedia of neuroscience.

[bib0016] Bhongade M.B., Prasad S., Jiloha R., Ray P., Mohapatra S., Koner B. (2015). Effect of psychological stress on fertility hormones and seminal quality in male partners of infertile couples. Andrologia.

[bib0017] Bowling, E. R. 2003. Sperm mobility in broiler breeders.

[bib0018] Darbandi M., Darbandi S., Agarwal A., Sengupta P., Durairajanayagam D., Henkel R., Sadeghi M.R. (2018). Reactive oxygen species and male reproductive hormones. Reprod. Biol. Endocrinol..

[bib0019] Darmawan A., Hermana W., Suci D.M., Mutia R., Jayanegara A., Ozturk E. (2022). Dietary phytogenic extracts favorably influence productivity, egg quality, blood constituents, antioxidant and immunological parameters of laying hens: a meta-analysis. Animals.

[bib0020] Du J.-H., Xu M.-Y., Wang Y., Lei Z., Yu Z., Li M.-Y. (2022). Evaluation of Taraxacum mongolicum flavonoids in diets for Channa argus based on growth performance, immune responses, apoptosis and antioxidant defense system under lipopolysaccharide stress. Fish Shellfish Immunol..

[bib0021] El Basuini M.F., El-Hais A.M., Dawood M.A., Abou-Zeid A.E.-S., EL-Damrawy S.Z., Khalafalla M.M.E.-S., Koshio S., Ishikawa M., Dossou S. (2016). Effect of different levels of dietary copper nanoparticles and copper sulfate on growth performance, blood biochemical profiles, antioxidant status and immune response of red sea bream (Pagrus major). Aquaculture.

[bib0022] Ems T., St Lucia K., Huecker M.R. (2017). StatPearls.

[bib0023] Fallah A., Mohammad-Hasani A., Colagar A.H. (2018). Zinc is an essential element for male fertility: a review of Zn roles in men’s health, germination, sperm quality, and fertilization. J. Reprod. Infertil..

[bib0024] Fouad A.M., El-Senousey H.K., Ruan D., Xia W., Chen W., Wang S., Zheng C. (2020). Nutritional modulation of fertility in male poultry. Poult. Sci..

[bib0025] Gęgotek A., Skrzydlewska E. (2022). Antioxidative and anti-inflammatory activity of ascorbic acid. Antioxidants.

[bib0026] Ghadimi M., Najafi A., Sharifi S.D., Mohammadi-Sangcheshmeh A., Mehr M.R.-A. (2024). Effects of dietary Moringa oleifera leaf extract on semen characteristics, fertility, and hatchability in aged broiler breeder roosters. Poult. Sci..

[bib70] Hcini K., Ben F., Bendhifi Z., Kahlaoui S., Stambouli-Essassi S. (2025). Polyphenolic profile, total phenolic content and antioxidant activity of Tunisian cultivated sage (Salvia officinalis L.) extracts. J. Agric. Food Sci. Biotechnol..

[bib0027] Hosseini H., Esmaeili N., Sepehr A., Zare M., Rombenso A., Badierah R., Redwan E.M. (2022). Does supplementing laying hen diets with a herb mixture mitigate the negative impacts of excessive inclusion of extruded flaxseed?. Anim. Biosci..

[bib0028] JAMOVI (2023). The jamovi project (2023). Jamovi. Comput. Softw..

[bib0029] Koyama T., Terhzaz S., Naseem M.T., Nagy S., Rewitz K., Dow J.A., Davies S.A., Halberg K.V. (2021). A nutrient-responsive hormonal circuit mediates an inter-tissue program regulating metabolic homeostasis in adult Drosophila. Nat. Commun..

[bib0030] Leão R., Castro F., Xavier P., Vaz D., Grázia J., Baião N., Avelar G., Marques A. (2017). Comb, cloaca and feet scores and testis morphometry in male broiler breeders at two different ages. Arq. Bras. Med. Vet. Zootec..

[bib0031] Li Y., Wang K., Li C. (2024). Oxidative stress in poultry and the therapeutic role of herbal medicine in intestinal health. Antioxidants.

[bib0032] Liang W., He Y., Zhu T., Zhang B., Liu S., Guo H., Liu P., Liu H., Li D., Kang X. (2024). Dietary restriction promote sperm remodeling in aged roosters based on transcriptome analysis. BMC Genom..

[bib0033] Madesh M., Yan J., Jinan G., Hu P., Kim I.H., Liu H.-Y., Ennab W., Jha R., Cai D. (2025). Phytogenics in swine nutrition and their effects on growth performance, nutrient utilization, gut health, and meat quality: a review. Stress Biol..

[bib0034] McDaniel C., Hannah J., Parker H., Smith T., Schultz C., Zumwalt C. (1998). Use of a sperm analyzer for evaluating broiler breeder males. 1. Effects of altering sperm quality and quantity on the sperm motility index. Poult. Sci..

[bib0035] Mehri M., Jalilvand G., Ghazaghi M., Mahdavi A.-H., Kasmani F.B. (2013). Estimation of optimal lysine in quail chicks during the second and third weeks of age. It. J. Anim. Sci..

[bib0036] Mendiratta S., Qu Z.-c., M J. (May. 1998). Erythrocyte ascorbate recycling: antioxidant effects in blood. Free Radic. Biol. Med..

[bib0037] Mfoundou J.D.L., Guo Y., Yan Z., Wang X. (2022). Morpho-histology and morphometry of chicken testes and seminiferous tubules among yellow-feathered broilers of different ages. Vet. Sci..

[bib0038] Mnisi C.M., Mlambo V., Gila A., Matabane A.N., Mthiyane D.M., Kumanda C., Manyeula F., Gajana C.S. (2022). Antioxidant and antimicrobial properties of selected phytogenics for sustainable poultry production. Appl. Sci..

[bib0039] Moon S.-G., Lee S.-K., Lee W.-D., Niu K.-M., Hwang W.-U., Oh J.-S., Kothari D., Kim S.-K. (2020). Effect of dietary supplementation of a phytogenic blend containing Schisandra chinensis, Pinus densiflora, and Allium tuberosum on productivity, egg quality, and health parameters in laying hens. Anim. Biosci..

[bib0040] Murawski M., Saczko J., Marcinkowska A., Chwiłkowska A., Gryboś M., Banaś T. (2007). Evaluation of superoxide dismutase activity and its impact on semen quality parameters of infertile men. Folia Histochem. Cytobiol..

[bib0041] Najafi A., Taheri R.A., Mehdipour M., Martínez-Pastor F., Rouhollahi A.A., Nourani M.R. (2019). Improvement of post-thawed sperm quality in broiler breeder roosters by ellagic acid-loaded liposomes. Poult. Sci..

[bib0042] Nemati Z., Dehgani P., Karimi A., Amirdahri S., Kianifard D. (2023). Effects of ginger (Zingiber officinale) supplementation on testicular histology, semen characteristic, blood plasma parameters and reproductive performance in aged broiler breeder roosters. J. Anim. Physiolo. Anim. Nutr..

[bib0043] Ngcobo J.N., Ramukhithi F.V., Nephawe K.A., Mpofu T.J., Chokoe T.C., Nedambale T.L. (2021). Flaxseed oil as a source of omega n-3 fatty acids to improve semen quality from livestock animals: a review. Animals.

[bib0044] Pan X., Köberle M., Ghashghaeinia M. (2024). Vitamin C-dependent uptake of non-heme iron by enterocytes, its impact on erythropoiesis and redox capacity of human erythrocytes. Antioxidants.

[bib0045] Pasqualotto F.F., Sharma R.K., Potts J.M., Nelson D.R., Thomas Jr A.J., Agarwal A. (2000). Seminal oxidative stress in patients with chronic prostatitis. Urology.

[bib0046] Peña F., Valencia S., Tereucán G., Nahuelcura J., Jiménez-Aspee F., Cornejo P., Ruiz A. (2023). Bioactive compounds and antioxidant activity in the fruit of rosehip (Rosa canina L. and Rosa rubiginosa L.). Molecules.

[bib0047] Poulios E., Giaginis C., Vasios G.K. (2020). Current state of the art on the antioxidant activity of sage (Salvia spp.) and its bioactive components. Planta Medica.

[bib0048] Powell S.R. (2000). The antioxidant properties of zinc. J. Nutr..

[bib0049] Ratchamak R., Authaida S., Koedkanmark T., Boonkum W., Semaming Y., Chankitisakul V. (2023). Supplementation of freezing medium with Ginseng improves rooster sperm quality and fertility relative to free radicals and antioxidant enzymes. Animals.

[bib0050] Ratchamak R., Authaida S., Koedkanmark T., Boonkum W., Semaming Y., Chankitisakul V. (2024). Dietary supplementation with ginseng extract enhances testicular function, semen preservation, and fertility rate of mature and aging Thai native roosters. Theriogenology.

[bib0051] Rizvi S., Raza S.T., Ahmed F., Ahmad A., Abbas S., Mahdi F. (2014). The role of vitamin E in human health and some diseases. Sultan Qaboos Univ. Med. J..

[bib0053] Sarabia Fragoso J., Díaz M.P., Abad Moreno J., Casanovas Infesta P., Rodriguez-Bertos A., Barger K. (2013). Relationships between fertility and some parameters in male broiler breeders (body and testicular weight, histology and immunohistochemistry of testes, spermatogenesis and hormonal levels). Reprod. Domest. Anim..

[bib0054] SAS (2002).

[bib0055] Shan T., Dai P., Zhu P., Chen L., Wu W., Li Y., Li C. (2017). Effect of an organic trace mineral premix on the semen quality, testicular morphology and gene expression related to testosterone synthesis of male broiler breeders. Rev. Bras. Cienc. Avic..

[bib0056] Stansbury J., Saunders P.R., Zampieron E.R., Winston D. (2013). The treatment of liver disease with botanical agents. J. Restor. Med..

[bib0057] Sun L., He M., Wu C., Zhang S., Dai J., Zhang D. (2021). Beneficial influence of soybean lecithin nanoparticles on rooster frozen–thawed semen quality and fertility. Animals.

[bib0058] Surai P., Fisinin V. (2014). Selenium in poultry breeder nutrition: an update. Anim. Feed Sci. Technol..

[bib0059] Surai P.F. (2018).

[bib0060] Surai P.F. (2020). Antioxidants in poultry nutrition and reproduction: an update. Antioxidants.

[bib0061] Thiam M., Barreto Sánchez A.L., Zhang J., Zheng M., Wen J., Zhao G., Wang Q. (2021). Association of heterophil/lymphocyte ratio with intestinal barrier function and immune response to salmonella enteritidis infection in chicken. Animals.

[bib0062] Thiam M., Wang Q., Barreto Sánchez A.L., Zhang J., Ding J., Wang H., Zhang Q., Zhang N., Wang J., Li Q. (2022). Heterophil/lymphocyte ratio level modulates Salmonella resistance, cecal microbiota composition and functional capacity in infected chicken. Front. Immunol..

[bib0063] Tinggi U. (2008). Selenium: its role as antioxidant in human health. Environ. Health Prev. Med..

[bib0064] Virant-Klun I., Imamovic-Kumalic S., Pinter B. (2022). From oxidative stress to male infertility: review of the associations of endocrine-disrupting chemicals (bisphenols, phthalates, and parabens) with human semen quality. Antioxidants.

[bib0065] Wang J., Zhang J., Wang Q., Zhang Q., Thiam M., Zhu B., Ying F., Elsharkawy M.S., Zheng M., Wen J. (2023). A heterophil/lymphocyte-selected population reveals the phosphatase PTPRJ is associated with immune defense in chickens. Commun. Biol..

[bib0066] Wilson J., Krista L., McDaniel G., Sutton C. (1988). Correlation of broiler breeder male semen production and testes morphology. Poult. Sci..

[bib0067] Windisch W., Schedle K., Plitzner C., Kroismayr A. (2008). Use of phytogenic products as feed additives for swine and poultry. J. Anim. Sci..

[bib0068] Xiong Y., Xiong Y., Zhou S., Sun Y., Zhao Y., Ren X., Zhang Y., Zhang N. (2017). Vitamin C and E supplements enhance the antioxidant capacity of erythrocytes obtained from aged rats. Rejuvenation Res..

[bib0069] Zhao W., Adjei M., Zhang Z., Yuan Z., Cisang Z., Song T. (2023). The role of GnRH in Tibetan male sheep and goat reproduction. Reprod. Domest. Anim..

